# Structure and domain dynamics of human lactoferrin in solution and the influence of Fe(III)-ion ligand binding

**DOI:** 10.1186/s13628-016-0032-3

**Published:** 2016-11-04

**Authors:** Clemens Sill, Ralf Biehl, Bernd Hoffmann, Aurel Radulescu, Marie-Sousai Appavou, Bela Farago, Rudolf Merkel, Dieter Richter

**Affiliations:** 1 JCNS-1 & ICS-1, Forschungszentrum Jülich GmbH, Leo-Brandt Strasse, 52425 Jülich, Germany; 2 ICS-7, Forschungszentrum Jülich GmbH, Leo-Brandt Strasse, 52425 Jülich, Germany; 3 JCNS-MLZ, Forschungszentrum Jülich GmbH Outstation at MLZ, Lichtenbergstraße, 1 85747 Garching, Germany; 4 Institute Laue-Langevin, CS 20156, 38042 Grenoble, France

**Keywords:** Protein, Domain dynamics, Steric suppression, Neutron spinecho spectroscopy, Small angle neutron scattering

## Abstract

**Background:**

Human lactoferrin is an iron-binding protein of the innate immune system consisting of two connected lobes, each with a binding site located in a cleft. The clefts in each lobe undergo a hinge movement from open to close when Fe^3+^ is present in the solution and can be bound. The binding mechanism was assumed to relate on thermal domain fluctuations of the cleft domains prior to binding. We used Small Angle Neutron Scattering and Neutron Spin Echo Spectroscopy to determine the lactoferrin structure and domain dynamics in solution.

**Results:**

When Fe^3+^ is present in solution interparticle interactions change from repulsive to attractive in conjunction with emerging metas aggregates, which are not observed without Fe^3+^. The protein form factor shows the expected change due to lobe closing if Fe^3+^ is present. The dominating motions of internal domain dynamics with relaxation times in the 30–50 ns range show strong bending and stretching modes with a steric suppressed torsion, but are almost independent of the cleft conformation. Thermally driven cleft closing motions of relevant amplitude are not observed if the cleft is open.

**Conclusion:**

The Fe^3+^ binding mechanism is not related to thermal equilibrium fluctuations closing the cleft. A likely explanation may be that upon entering the cleft the iron ion first binds weakly which destabilizes and softens the hinge region and enables large fluctuations that then close the cleft resulting in the final formation of the stable iron binding site and, at the same time, stable closed conformation.

**Electronic supplementary material:**

The online version of this article (doi:10.1186/s13628-016-0032-3) contains supplementary material, which is available to authorized users.

## Background

Lactoferrin (Lf) is an iron-binding globular protein found in milk and many other secretory fluids like saliva, tears and mucosal secretions of bronchial, nasal, lachrymal, and genitourinary passages of the body [1]. Because of its antibacterial, antiviral and antifungal activity it belongs to the innate immune system. Human lactoferrin (hLf) is a monomeric protein consisting of a single polypeptide chain of 691 amino acid residues and shares the main structure with other proteins from the transferrin family. The transferrin family proteins have two main lobes connected by a short linker (for hLf an alpha helix) and each of the lobes possesses a binding site for an iron ion located in a cleft between two domains [2, 3]. The C-terminal lobe and the N-terminal lobe share a sequence identity of about 40 % and the binding site is equally structured in all transferrins of higher organisms.

The binding of Fe^3+^-ions is accompanied with an intense red coloration of hLf. Iron is bound reversibly, but with a very high binding affinity (K ~ 10^22^ M, [3, 4]) which is about 300 times stronger than the binding affinity of the main iron transport protein transferrin, with which it shares 60 % sequence identity [5, 6]. The high binding affinity is the cause for the main antibacterial activity as it removes the essential iron for bacteria growth beside a variety of other functions [3, 7].

**Fig. 1:**
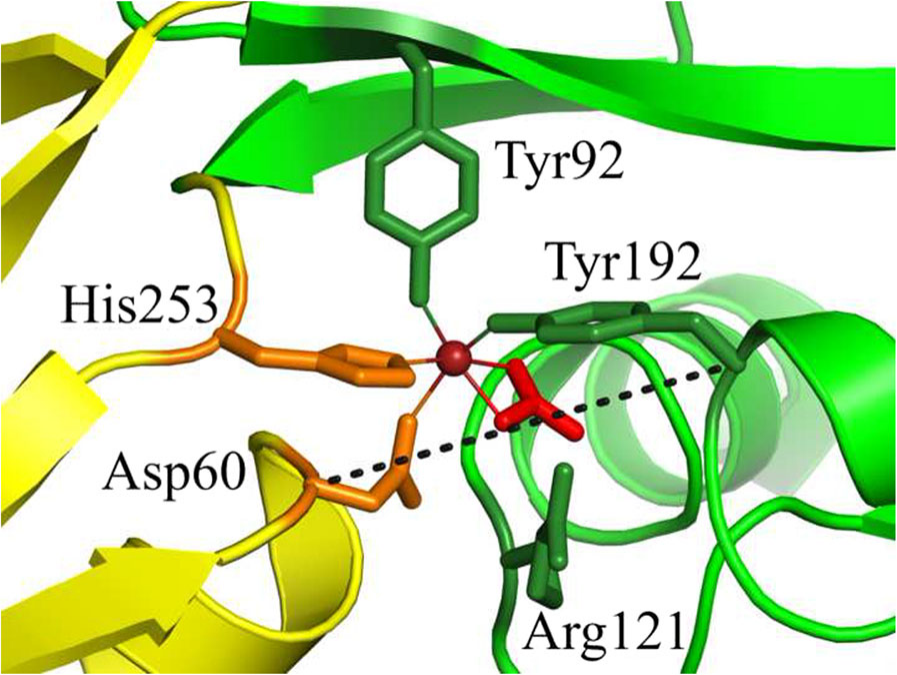
N-terminal lobe binding site of lactoferrin (PDB 1b0l) showing the iron (*red* sphere) coordinated with CO_3_^2−^ (*red* triskelion) and the surrounding amino acids from the cleft domains (*yellow* and *green*). The hinge is found in the region between yellow and green colored structural elements in the upper left. The width of the cleft can be characterized by the C_α_-distance between Asp60 and Tyr192 with 11.3 Å (broken line), which becomes 19.3 Å in the open configuration. Arg121 is located at the helix 5 N-termini

The binding site in the cleft of each lobe is built from a histidine, an aspartic acid and two tyrosines with the capacity to bind a Fe^3+^ ion together with an arginine-bound CO_3_^2−^ anion, as shown in Fig. 1 [8]. The charges of the iron and the anion are balanced by opposite charges of the protein and the anion causes the dependence of iron release on pH [3]. Both sides of the cleft contribute to the binding and are cross-linked in this way. The two lobes are similar but not identical and a slight difference in binding affinity and pH dependence is reported [9]. Lf has the ability to bind iron even at low pH, which is important in case of inflammation, where pH can drop down to pH 4.5 due to metabolic activity of bacteria. In this case, Lf can even bind iron released from transferrin [10]. The iron binding seems to be unaffected by glycosylation of Lf [11, 12].

**Fig. 2:**
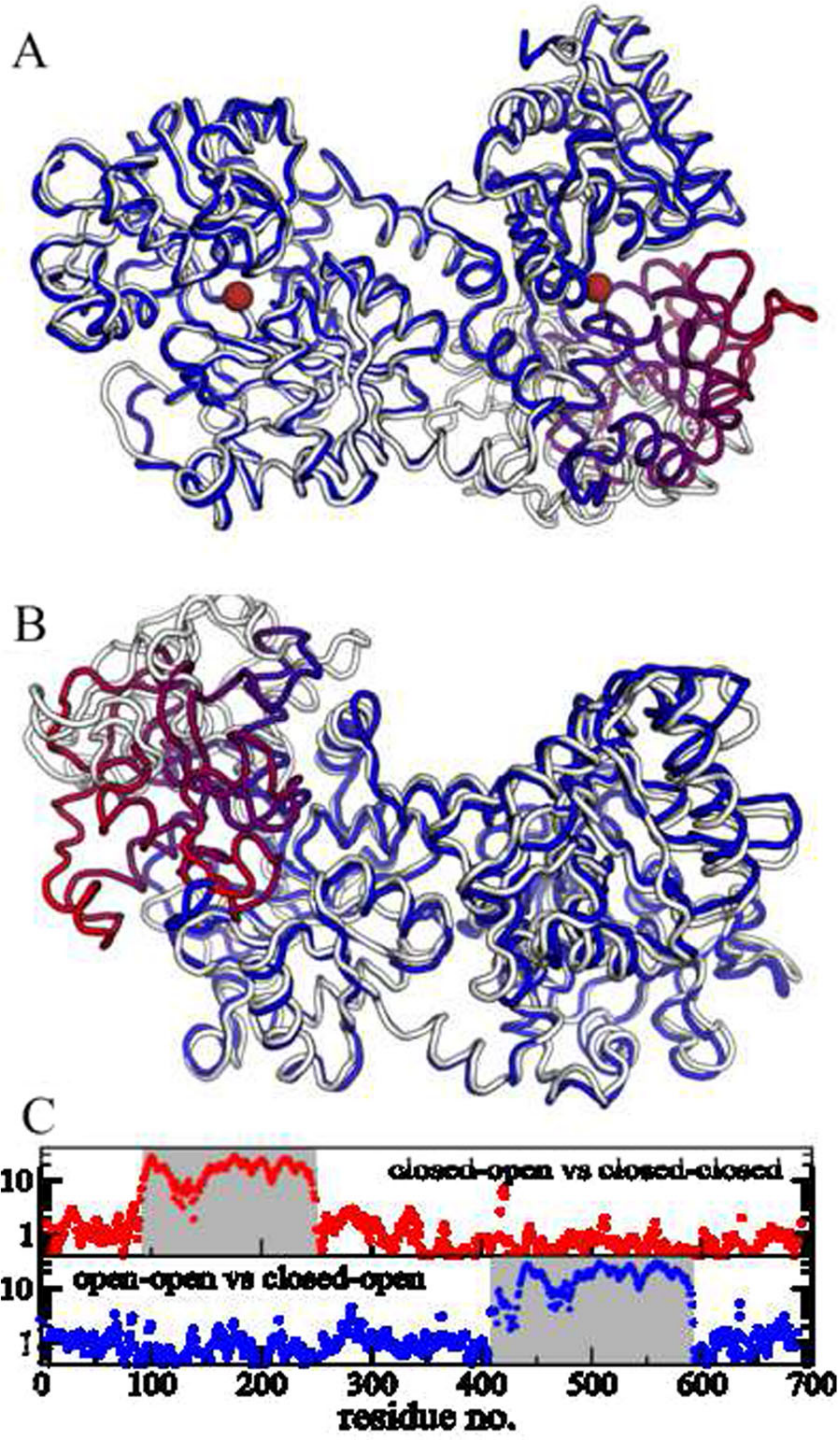
Structure of hLf with two main lobes (C-terminal left and N-terminal right) and a cleft between domains in each lobe. **a**) Comparison of closed-open vs. closed-closed structure with the iron ions (red spheres) marking the binding sites. Closed-open is shown in white-black-bordered, closed-closed is color-coded by distance of the C_α_-atoms between the structures: from 0 Å (blue) to 32 Å (red). **b**) The same representation for open-open vs. closed-open structure. **c**) Shows the translation distances of C_α_-atoms between the respective structures. Only about 1/4 of the residues show a large displacement when closing one cleft

Lf was one of the first proteins where crystallography showed large-scale conformational changes, as described by Anderson et al. [13]. In a comparison of the crystal structures of iron-saturated Lf and apo-Lf it was shown that the N-terminal binding cleft is open when unliganded and closed when occupied, both configurations differ in a 54° rigid body rotation of one domain in a lobe as shown in Fig. 2. However, the C-terminal binding cleft was found to be closed in both cases. As likely explanation an influence of the crystal packing was mentioned.

An overview of the crystal structures of human lactoferrin available today in the protein data bank shows that the issue of the C-terminal binding cleft remains unresolved. The PDB structures can be classified in two groups: both binding clefts are closed (“closed-closed”): 1b0l (recombinant Fe(III)_2_-Lf), 1fck (Ce(IV)_2_-Lf), 1lfg (apo-Lf), 1lfi (Cu(II)_2_-Lf), 1sqy (Cu(II)_2_-Lf). The structures in the second group have a closed C-terminal cleft and an open N-terminal cleft (“closed-open”): 1lfh (apo-Lf), 1cb6 (apo-Lf). The distances of corresponding C_α_-atoms for all PDB structures in each group deviate only for 5 out of 691 residues by more than 1.5 Å, which is the typical spatial resolution of crystallography, showing that only two major conformations were observed.

It has been suggested from small angle X-ray scattering, that both binding clefts of iron free Lf are open in solution [14], however at present there is no such structure available in the protein data bank.

Tight iron binding by domain closure with the ability for release in an open configuration requires a mechanism of configurational change.

Relating to a binding mechanism it was suggested by Baker et al. that the iron binds in the open configuration first to one domain and then the cleft closes leading to an attractive interaction of the iron ion with both sides of the binding site, thus locking the closed conformation [15]. Gerstein and Baker assumed that in the open configuration thermal fluctuations are sufficient to close the cleft and if an iron was bound, the closed form is stabilized [2, 11, 16]. Gerstein assumes a similar energy between open and closed states, moreover Baker constrains to transient visits of the closed conformation - which contradicts the similar energy assumption, assuming a Boltzmann factor- to justify solution scattering results of Grossmann et al. [14]. In this context electrostatic interaction cannot cause the domain closure from the open configuration, because the distance of iron to the other domain would be too large (>10 Å, equivalent to 19.3 Å for the Cα distance in Fig. 1). Iron release is then triggered mainly by pH change through a destabilization of the cleft.

Recently Neutron Spin Echo Spectroscopy (NSE) was used to examine the domain dynamics of multidomain proteins as phosphoglycerate kinase and alcohol dehydrogenase [17, 18]. With its space-time resolution on the molecular scale, NSE is sensitive to internal protein dynamics on the nanometer and nanosecond scale. A detailed normal mode analysis allows the description of main domain movements and a determination of their motion amplitudes and timescales [19].

The goal of this work is to investigate the mechanism of domain closure induced by iron binding in solution. According to the proposed mechanism domain movements should be observed in the open configuration without bound iron, while with bound iron the corresponding movements should be suppressed. From the comparison between both, a picture of the underlying mechanisms will be developed.

## Results and discussion

### Structure: dependence of conformation on iron content

The small angle neutron scattering (SANS) of proteins in solution depends on the protein form factor F(Q) and on the spatial arrangement between proteins comprised in the structure factor S(Q). Both can be extracted from measurements at different concentrations as described in the Methods section. The form factor F(Q) contains the information about the shape of the protein. F(Q) can be calculated from the atomic coordinates of a protein structure e.g. from the PDB. This allows a comparison of a given three-dimensional atomistic protein structure model with the experimental findings.

For this, we used the 1b0l PDB structure [20] for the closed-closed and the 1lfh PDB structure [21] for the closed-open conformation. Since there is no structure with both binding clefts open available, we created an appropriate homologue model with the help of the SWISS-MODEL [22, 23] homologue modeling server as open-open structure. As input we used the amino acid sequence of 1lfh.pdb and the structure of 1dtz.pdb (camelus dromedarius apo-Lf [24]) as template, which both share a 74 % sequence similarity.

Figure 2a and b show pairwise comparison of the three structures. The ribbon representation as well as the displacement histograms in Fig. 2c show that the opening or closing of a cleft involves only about 1/4 of the amino acids: only one half of the cleft shows large displacements, the other half of this cleft and the entire other cleft stays virtually untouched.

**Fig. 3:**
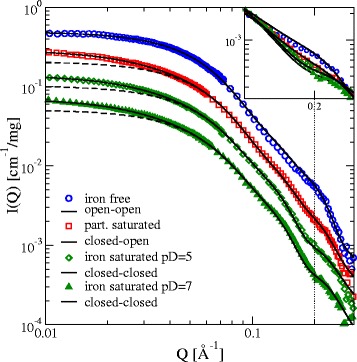
Extrapolated form factors per unit mass from the experiments (symbols), consecutively scaled down by a factor of 2 for better visibility (iron free not scaled). The dashed lines show the best fitting calculated form factor from the structure models. The solid lines include a Guinier function in the calculated form factor to account for small protein aggregates, which results in a much better agreement with the experimental data at low Q. The inset magnifies the Q-range around 0.2 Å^−1^ (without scaling), where the conformational change is most prominent. Errors are below 2 %. A concentration series of iron saturated pD5 and iron free samples are given in Additional file 1 S2a-d

The form factors F(Q) extracted from the SANS experiments on lactoferrin are shown in Fig. 3. The comparison with calculated form factors from the structure models shows good agreement at higher Q. The radius of gyration for iron free Lf 29.1(±0.2) Å agrees well with 29.0(±0.2) Å for the open-open model showing an excellent agreement and no sign of present aggregates. We observe notable differences in the forward scattering I(Q = 0) for the iron containing samples. This is visible also when looking at the radius of gyration R_g_ from the experiment and the closest matching model structures: iron saturated Lf (pD = 7 and pD = 5) with 31.2 respectively 31.4 (±0.2) Å is significantly bigger than the closed-closed model with 28.5 (±0.2) Å. The same is the case for the partially saturated Lf (31.2 ± 0.2 Å vs. 27.8 ± 0.2 Å closed-open).

This discrepancy can be explained, if a mixture of single proteins with small protein aggregates is assumed. This is modeled by adding a Guinier function to the calculated protein form factor: F_tot_(Q) = I_0,hLf_F_calc_(Q) + I_0,agg_exp(−Q^2^R_agg_^2^/3) (see Materials and Methods). The parameters I_0,agg_ and R_agg_ were fitted and with I_0,agg_/I_0,hLf_ = 0.3-0.33 and R_agg_ = 63-68 Å the experimental F(Q) is very well described at low Q. The Guinier description of the aggregates also allows to judge their contribution to the scattering intensity at higher Q relevant for later NSE measurements: for Q > 0.055 Å^−1^ the aggregate signal is less than 1 % of the monomer signal and negligible at higher Q (see in Additional file 1: Figure S1).

It is not possible to modify the three conformations of the monomeric molecule (e.g. by means of Normal Mode Analysis) in a way that the resulting form factor explains the discrepancy in R_g_ and matches the experimental F(Q) at higher Q at the same time. The reason for the aggregates is an attractive interaction as will be described later. It should be noted that the appearance of aggregates in the zero-extrapolated form factor implies that the aggregates don’t vanish with lowered concentration as may be expected. This might be a result from attractive interaction leading to a phase separation with high-density clusters even for low overall concentrations.

Speaking of the high Q range, the difference between the conformations is predominantly visible around Q = 0.2 Å^−1^, where the scattering intensity decreases in the order iron free, partially iron saturated and iron saturated. A comparison of the calculated form factors with the measurements shows: the open-open model agrees perfectly with iron free Lf, the closed-open model matches the partially iron saturated Lf and the closed-closed model shows great similarity with the iron saturated Lf at both pD values. In case of the partially and fully iron saturated Lf, fractions of possible open and closed structures are averaged in order to get even better agreement to the experiments. The best fitting fractions of this refinement are listed in Table 1 and results are shown in Fig. 3. It needs to be clarified that the modifications take three contributions to a changed form factor together. (i) The iron saturation and removal may be not complete and we need to include e.g. for the iron saturated sample a contribution of iron free configurations because of incomplete saturation. (ii) The assumption that the molecule exhibits the same conformation in solution and in the crystal. The packing into a periodic lattice may influence molecular structures and could change configurations in some extend compared to the solution structure. (iii) The assumption of dynamical cleft closure in solution as a binding mechanism includes, that the form factor is an ensemble average over the distribution of different configurations rather than a single conformation. In particular the equal distribution between open-open and closed-closed configurations (in a two state model), the equal distribution of interpolated configurations between open and closed (dynamical cleft closure) and the open-closed configuration look quite similar (see Additional file 1: Figure S2e). Discrimination between the different contributions is not possible within static measurements.

**Table 1:** Fractions of the different structures to the best fitting structures

Sample name	Open-open	Closed-open	Closed-closed
iron free	100 %	-	-
partially saturated	37 %	47 %	15 %
iron saturated pD = 5	8 %	6 %	86 %
iron saturated pD = 7	0 %	16 %	83 %

The previously assumed open-open conformation of apo-hLf is modeled with homology and experimentally verified in the iron free case. Contributions from iron bound states, crystal packing and dynamical ensembles seem to be negligible. The iron saturated samples seem to contain a portion of not completely closed structures due to incomplete saturation. The partially saturated sample resembles equilibrium between all 3 states with a probable majority in the closed-open structure. Overall the dependence of the Lf structure on the iron binding state is reproduced and the observed deviations correspond to the measured iron saturation of 5 %, 30 % and 85 % (see Methods). For all conditions except the iron free state we find small meta-stable aggregates of about 10 monomers in size (R_agg_/R_hLf_ =2.2–2.4). The metastable character is due to attractive interactions (see below discussion about the structure factor). Regarding equilibrium fluctuations between open and closed state in a two state system with a negligible energy difference would result in 50 % occupation of both states, resulting in a form factor similar to the closed-open structure as average between open-open and closed-closed. Assuming an energy difference of 1 *kT* (2 *kT*) results in 25 % (11 %) occupation of the higher energy state according to a Boltzmann distribution. Assuming that we can resolve about 10 % change in the form factor at the prominent Q = 0.2 Å^−1^ position, we may conclude that the energy difference needs to be larger than 2 *kT* to be compatible to the observation of negligible changes compared to the open-open configuration in the iron free sample*.*

### Structure factor: interparticle interaction

The structure factor S(Q,c) contains information about the spatial distribution of the proteins in the solvent and therefore about the interaction potential between the molecules. It can be calculated from the scattering intensity I(Q) and the form factor F(Q): S(Q,c) = I(Q)/(c F(Q)), which is strictly valid only for identical spherical particles. For asymmetric particles or polydisperse systems an effective structure factor is obtained neglecting possible orientational correlations [25, 26]. Typically, S(Q,c) is equal to 1 for infinite dilution as well as for high Q, where the probed length scale is much smaller than the distance between proteins. In both cases, there is no interparticle correlation measured, thus S(Q,c) = 1. The experimental form factor from extrapolation contains contribution from aggregates. These aggregates are always present and also depend on concentration. For the structure factor calculation they need to be taken into account to prevent artifacts in the structure factor. This is done best by using the experimental form factor from extrapolation to c = 0 for S(Q,c) calculation and regarding the resulting structure factor as an effective structure factor between monomers and aggregates. As the number density of the aggregates is more than a factor of 300 smaller than the monomer number density (see Additional file 1: Figure S1) contributing aggregate-aggregate or aggregate-monomer partial structure factors should be close to one and contribute less due to the low cluster concentration. Thus the structure factor contains mainly information about monomer-monomer interactions. Nevertheless, the effective structure factor might contain contributions of cluster number density or size variations with concentration and smaller contribution from the partial structure factors related to cluster-cluster and cluster-monomer interactions.

**Fig. 4:**
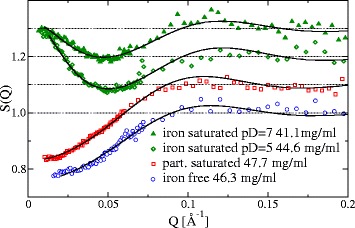
Experimental structure factors S(Q,c) (symbols) for the highest concentrations of c ≈ 50 mg/ml. Curves are displaced successively by adding 0.1 for better visibility. The partially saturated and iron free samples decline towards Q = 0 and can be well described by a hard-sphere model (solid lines: Percus-Yevick structure factor fit). Both fully iron saturated samples show an upturn at small Q, indicating attractive behavior. This can be modeled as hard spheres with Debye-Hückel like attraction (solid lines: Hayter-Penfold structure factor fit)

In Fig. 4, the experimental S(Q,c) of the different Lf samples are shown for the highest concentration of about 50 mg/ml, where the structure factor shows the strongest variation. Two different behaviors at low Q can be observed: the iron free and partially saturated Lf show a decline towards Q = 0, whereas both iron saturated samples show a minimum around Q = 0.05 Å^−1^ and an increase for smaller Q. The decline towards small Q is typical for repulsive interaction, whereas the increase is characteristic for attractive behavior. The present aggregates do not cause this behavior as can be seen for the partial saturated sample showing a purely repulsive behavior. The structure factors with a decline towards Q = 0 are well described by a hard-sphere interaction and the associated Percus-Yevick structure factor [27–29]. The fitted hard-sphere radius R_HS_ is 25.6 Å for iron free Lf and 26.6 Å for partially saturated Lf. Both values are only slightly smaller than the radius of 27 Å of a sphere with equivalent molecular volume of about 85000 Å^3^ (calculated with CHIMERA [30]).

The attractive S(Q,c) of both iron saturated Lf samples can be modeled by adding a Debye-Hückel type short-range interaction to hard spheres, similar to a screened Coulomb potential: *U*(*r*) = *γβ* ^− 1^ *e* ^*kx*^ *x* ^− 1^ for x > 1 (outside the sphere) with x = r/2R_HS_, β^−1^ = k_B_T, k = κ2R_HS_. The structure factor for this system was derived by Hayter and Penfold in a mean spherical approximation [31] and has four parameters: radius R_HS_, screening length κ^−1^, interaction range γ and volume fraction Φ. This number of parameters makes necessary to fix one of them in order to determine the others by a fitting procedure. We fix R_HS_ to 25 Å, a value close to the results of the pure hard-sphere model, and obtain a screening length κ^−1^ of 21.4 Å (pD = 5) resp. 19.6 Å (pD = 7) and a contact potential U(2R_HS_) = γe^-k^ of −0.88 k_B_T (pD = 5) resp. −0.72 k_B_T (pD = 7). These values may be biased through the choice of R_HS_, but they give an impression of the interaction strength and range. Short range attraction with weak long range coulomb repulsion allows formation of equilibrium clusters of several monomers as was shown by Stradner et al. [32]. This is a likely explanation for the small, meta-stable aggregates that can be found in the form factor.

The pD value and the iron binding state of Lf can be excluded as a possible reason for the occurrence of the attractive interaction. However, an influence of free Fe^3+^-ions in solution is a likely explanation: If Fe^3+^-ions dock on the surface of the protein, the strong charge is capable to change the local surface-charge of the protein [33]. Strongly charged surface areas lead to a behavior similar to patchy colloids. This might induce an attractive component in the interaction [34–36], which may be dominant in our case. Similar attractive interactions were found by Zhang et al. [37] for bovine serum albumin (BSA) in the presence of Y^3+^-ions. The iron saturated, freeze-dried lactoferrin contains probably excess ferric salts from the saturation process. The iron chelator EDTA in the buffer solution of the iron free samples can bind the iron ions and remove this effect, which is consistent with the occurrence of attractive and repulsive behavior. To a lesser degree this holds also for the partially saturated sample.

### Dynamics: thermal shape fluctuations

Neutron Spinecho spectroscopy (NSE) is a quasielastic neutron scattering technique that measures temporal and spatial correlations between different scattering particles and from internal motions in the particles resulting in the normalized intermediate scattering function I(Q,t)/I(Q,0) (see Methods). The observed protein dynamics in solution comprises several processes on different length- and timescales. There is the self-diffusion of the single protein molecule, which includes translation and rotation. At low Q the resolvable length scale is bigger than the protein, the protein looks point-like and only translational diffusion is observed. With increasing Q the probed length scale decreases and allows resolving finer details. When the spatial resolution is high enough to resolve the shape of the protein and its deviations from spherical symmetry, rotational diffusion becomes observable. Large-scale internal dynamics like domain motions are typically visible on a similar length scale as rotation. These three processes (translation, rotation, internal dynamics) can occur on different timescales. At even shorter length- and timescales, dynamics of smaller parts of the protein down to atomic groups can be observed. At finite concentrations, the interactions between the protein molecules have to be taken into account. There are direct correlations, such as excluded volume or Coulomb interactions, and indirect i.e. solvent-mediated ones.

To determine the contribution of internal dynamics to the NSE signal, the other above-mentioned components must be known (see Methods). The single particle diffusion D_0_ for a rigid protein can be calculated from the protein structure by using the HYDROPRO software [38–40] for given temperature and solvent viscosity and is also verified by dynamic light scattering (DLS). The viscosities of the used pure buffer solutions and protein solutions are measured with a rolling ball viscometer (Lovis 2000, Anton Paar) and determine the hydrodynamic function. The measured structure factor is used to account for direct interactions.

**Fig. 5:**
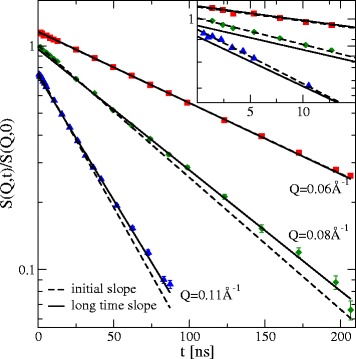
Semi logarithmic plot of the intermediate scattering function of iron free Lf (symbols) for three exemplary Q-values, displaced by a factor of 1.5 and 0.75 for better visibility. The dotted lines show the initial slope as obtained by D_1_ of a cumulant fit according to exp[−Q^2^(D_1_t + D_2_t^2^)], the solid lines show the long time slope from a single exponential fit for long times only, both extrapolated over the whole time range as single exponentials

In the case of low Q the intermediate scattering function is given as a single exponential: I(Q,t)/I(Q,0) = exp(−Q^2^Dt). This can be seen in Fig. 5, where I(Q,t)/I(Q,0) of iron free Lf is shown for three exemplary Q-values - full sets of measurements are shown in Additional file 1: Figure S3a-c. As expected for pure translational diffusion for the lowest Q = 0.06 Å^−1^ there is no difference between initial and long time slope. At higher Q, a clear distinction between initial and long time slope can be seen, of course depending on the covered time scale of the experiment. The long time D_eff_ refers in the following to the long time within the measurement and should not be confused with the long-time diffusion coefficient.

**Fig. 6:**
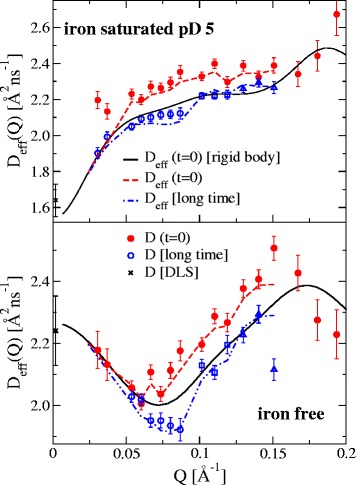
Effective diffusion coefficient D_eff_ of iron free Lf and iron saturated Lf at pD5: in the initial slope (*red* dots) and the long time behavior (*blue*: circles 50 ns < t < 200 ns, squares: 20 ns < t < 80 ns, triangle: 12 ns < t < 50 ns). The diffusion constant obtained from DLS (*black* cross) is shown as well as the expected rigid body diffusion D_eff_(Q) = D_0_(Q)H/S(Q,c) for the open-open and closed-closed model respectively in the initial slope (*black* lines). D_0_(Q) calculated from equ. 1. Broken lines show the slope analysis (initial and long time) of the fitted model, which agrees well with the experimental finding. The translational diffusion contribution D_T_(Q) = D_0_H_T_ for Q > 0.1 is 1.7 Å^2^/ns for iron free Lf and 1.74 Å^2^/ns for iron saturated Lf pD 5

The resulting effective diffusion coefficient values D_eff_ for t = 0 and for long times are shown in Fig. 6 in comparison to the rigid protein expectation (see Methods) for short times t → 0. A main difference between attractive interactions and purely repulsive interactions is visible at low Q comparing the iron free sample and the iron saturated sample. While the repulsive interaction with a strongly decreasing structure factor S(Q) leads to a strong increase at low Q according to D_eff_(Q) ~ D_0_/S(Q) the attractive structure factor forces a strong decrease in D_eff_(Q) even if the translational diffusion component D_0_*H_T_ for both has similar values. For small Q (<0.06 Å^−1^) there is no difference of initial and long time D_eff_, and the effective diffusion coefficient matches the values of DLS and those from the rigid body calculation, because there is no significant contribution from internal dynamics or rotational diffusion. For higher Q, the initial D_eff_ is faster than the rigid body, which is a clear sign of additional, faster, internal dynamics. The long time D_eff_ is below the rigid body prediction since rotational diffusion becomes visible in this Q range and is partially decayed at long times. For times much longer than the rotational correlation time (as 1/6D_R_ ≈ 120–140 ns) this will reach the limit of pure translational diffusion. The contribution from rotational diffusion to D_eff_(Q) in the initial slope for Q > 0.1 Å^2^/ns is about 20–30 % [41]. When the covered time scale of the experiment gets shorter (e.g. Q > 0.15 Å^−1^ with t_max_ < 50 ns) and the rotational diffusion is not yet decayed (Q > 0.1 Å^−1^) the long time diffusion shows a good reproduction of the expected rigid protein diffusion. At highest Q (>0.16 Å^−1^) the general behavior in the initial D_eff_ is continued, but the errors are too large for a later discrimination of internal amplitude and therefore omitted in the following.

The internal dynamics A(Q)e^-Γt^ in equ. 3 (see Methods) is able to model one additional internal relaxation process with characteristic time 1/Γ and magnitude A(Q) [17]. This model is fitted to all Q values of the experimental intermediate scattering function of one sample simultaneously, with the only free parameters 1/Γ and A(Q). As a check if all features of the experimental data are represented in this model after the least square fitting procedure, we perform the same analysis of initial and long time effective diffusion on the same time scales. This is plotted in Fig. 6 and shows good agreement with the experiment verifying the partial relaxation of rotational diffusion in midrange Q. (see Additional file 1 S4 for pD7)

**Fig. 7:**
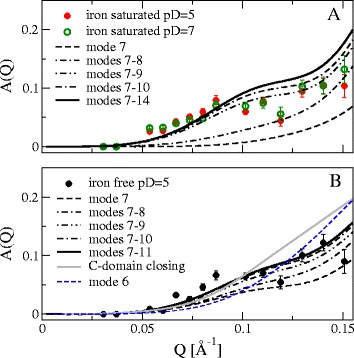
Experimental contribution A(Q) (symbols) of internal dynamics **a** for iron saturated (pD = 5 and 7) and **b** for iron free Lf (see equ. 3). The behavior is remarkably similar, except for 0.05 < Q < 0.075 Å^−1^, where the iron free Lf shows less dynamics than the others. In B the expected amplitude from mode 6 and for closure of the C-domain in the iron free Lf are shown. The lines show the modeling of internal dynamics (see equ. 4) based on the normal modes of deformation of the respective structure (closed-closed for iron saturated Lf, open-open for iron free Lf)

The resulting A(Q) is shown in Fig. 7 and the characteristic times 1/Γ together with the corresponding input parameters (diffusion and hydrodynamics) are listed in Table 2. The iron free and iron saturated Lf - both at pD = 5 - show the same characteristic time of about 50–55 ns, however the iron saturated Lf at pD = 7 is significantly faster with about 33 ns. The internal dynamics amplitude A(Q) is very similar for all three examined samples. Only the iron free Lf shows less dynamics for 0.05 < Q < 0.075 Å^−1^ than the iron saturated Lf. The change in pD from 5 to 7 has obviously no influence on the shape of A(Q) for iron saturated Lf.

**Table 2:** Input parameters D_T_, D_R_ and H_T_ for the fitted model, and obtained time scale 1/Γ of internal dynamics

	Iron free	Iron saturated	
	pD = 5	pD = 5	pD = 7
D_T_ [Å^2^/ns]	2.62	3.03	2.95
D_R_ [μs^−1^]	1.13	1.42	1.38
c [mg/ml]	47.2	43.6	40.8
H_T_	0.650	0.575	0.605
1/Γ [ns]	55.6 ± 13	50.9 ± 8.5	33.6 ± 5.1

The expected A(Q) for C-domain closure can be calculated from the difference in the configurations used as displacement vector and is shown in Fig. 7b with a dominant contribution at larger Q. Even partial closing of both binding clefts would significantly influence the observed A(Q) and generate a clear difference between iron free and iron saturated Lf, which is not observed.

To grasp the nature of the observed internal dynamics, we use elastic normal modes to describe thermal fluctuations of the protein structure and calculate the expected amplitude A(Q) (see Methods). Normal modes were obtained by an all-atom normal mode analysis with the Amber99 force field and are sorted in ascending order of their eigen frequencies. The slowest, softest modes come first. We assume thermal displacements from the equilibrium structure and over-damped relaxation due to solvent friction and protein internal friction.

Examining the contribution of the first non-trivial mode 6, we notice that the resulting A(Q) does not match the experimental A(Q) at all (see Fig. 7b). The calculated A(Q) of mode 6 with amplitude corresponding to the later modes 7–11 shows a steeper increase that starts at higher Q compared to the experiment. Mode 6 together with the higher order modes would thus lead to a strong overshot at larger Q in A(Q) suggesting a suppression of this mode leading to a minor negligible contribution of mode 6. A likely explanation for the suppression of mode 6 is a steric hindrance of the torsional motion represented by mode 6, which blocks larger displacements not taken into account by normal mode analysis at the energetic minimum. If we assume that mode 6 is strongly suppressed and we start the summation of modes at number 7, the resulting A(Q) is already very similar to the experiment with only 3–4 modes combined. It can be seen that the shoulder around 0.1 Å^−1^ evolves by adding normal mode 9. Adding more modes does not change the shape of A(Q) significantly, because the amplitudes for higher order modes become too small irrespective of their pattern. These modes describe more local deformations instead of large-scale domain motions of the lower modes.

**Fig. 8:**
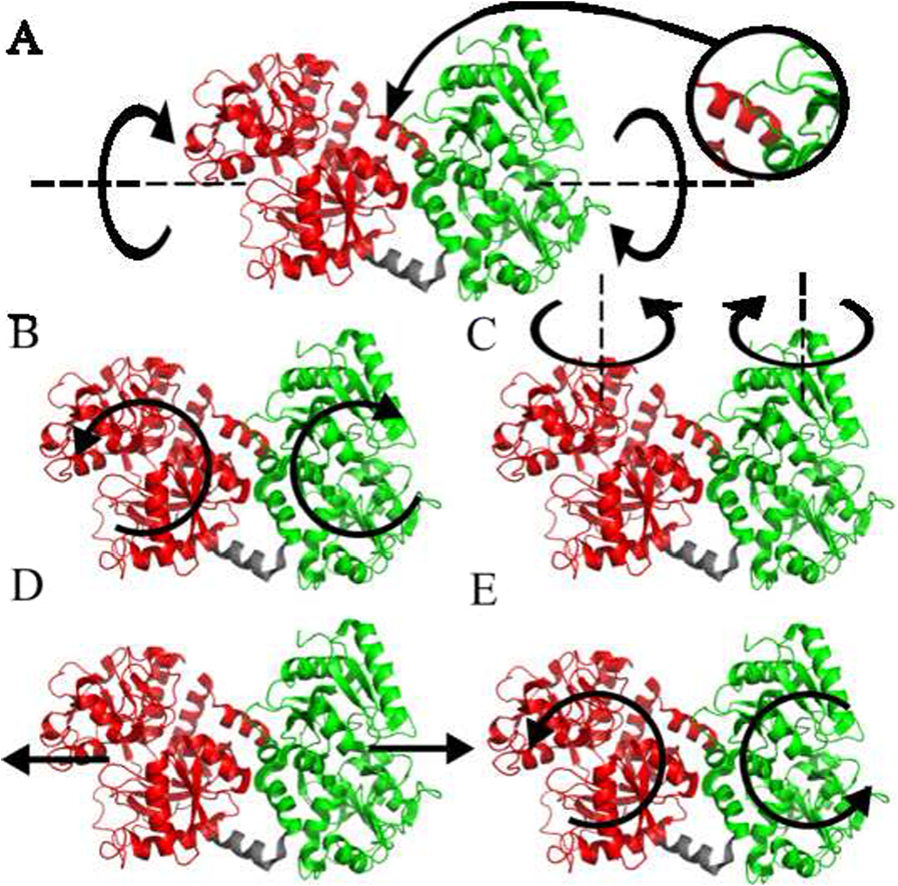
Motion patterns of the normal modes of deformation of Lf. **a**) Mode 6 is a torsion motion, which is suppressed by a steric hindrance (relevant section magnified). **b**) Mode 7 of open-open resp. mode 8 of closed-closed structure. **c**) Mode 8 of open-open resp. mode 7 of closed-closed structure. **d**) Mode 9 and **e**) mode 10 are the same for both structures

Interestingly, the dominant low frequency modes describe relative motions of the two main lobes (see Fig. 8). Mode 6 is a torsion motion between the lobes, mode 7, 8 and 10 are mainly bending modes, while mode 9 is a stretching mode that changes the distance between the lobes. Modes that open or close the binding clefts start at number 11 for the open-open structure and number 13 of the closed-closed structure. Mode 6 is hindered for larger torsions by an α-helix justifying the assumed suppression of this mode. Comparing mode 6 with the other modes, they are reasonable distinct in their displacements in the sense of orthogonality that we can assume a suppression of only mode 6 alone. Additional file 1: Table S5 lists the relative displacement amplitudes and the root mean square displacements for the fitted A(Q) for both structures (maximum displacement of 5.2 Å and 7.2 Å for mode 7). Beyond mode 10/11, the RMSD is on the same scale as a typical C-C bond length (~1.4 Å) and one can not speak of a large-scale internal motion per se, if the collective character of normal modes motions is ignored. For comparison, the conformational change from closed-closed to open-open has a RMSD of 12.3 Å with displacements in the cleft around 20–28 Å (see Fig. 2c). We conclude that the motion patterns of internal dynamics of Lf are independent of the iron binding state and are dominated by relative movements of the two lobes. Motions that open or close the binding clefts do not contribute significantly on the observed time and length scale, if they occur at all.

## Conclusions

The experiments confirm the closed-closed and closed-open structures for Fe(III)_2_-Lf and Fe(III)_1_-Lf respectively in solution. The overall shape, i.e. the position of the two lobes relative to each other, is basically the same as in the respective structure models based on x-ray crystallography. Apo-Lf showed to be consistent with a new homology model with both clefts open.

The structure factors of the protein solutions reveal that lactoferrin can show repulsive interaction (due to the excluded-volume as hard spheres without significant contributions from surface charges) or attractive interaction. Attractive interactions may be induced by binding of Fe^3+^-ions and local charge inversion at the surface building attractive patches [36].

A look at the internal dynamics shows that its magnitude A(Q) is independent of the investigated solvent conditions with repulsive or attractive interactions and independent of the iron binding state of lactoferrin. Solely the characteristic time scale depends on the pD-value of the solvent: as the pD mainly changes the charge distribution on the protein surface, an influence of the surface charge on the observed dynamics between the two lobes is suggested.

Modeling the motional pattern of the internal dynamics shows that not only the magnitude A(Q), but also the actual displacement pattern is independent from the examined conditions, including the suppression of the torsional mode 6 due to steric hindrance. The relevant normal modes show that the dominant motions are relative displacements of the two lobes that stretch and twist the protein. It is a remarkable result, that the conformational change related to the cleft opening has very little influence on the large-scale interdomain motion.

Regarding the closing mechanism for the binding of an iron ion, it was hypothesized that equilibrium fluctuations of the cleft between the two domains of a lobe bring the binding sites closer together [11, 16]. If thermal fluctuations on any timescale would notably include closing of the binding clefts, this would change the measured form factor significantly. Already the SANS results let the dynamical closing within thermal fluctuations appear unlikely or being connected with a larger energy difference between open and closed state (>2*kT*). Thermal fluctuations of the cleft around an open equilibrium configuration with relevant amplitudes are not observed within the NSE timescale as well. Therefore the conclusion is that both states seem to have a larger energy difference than assumed by Baker and Gerstein, which suppresses thermal fluctuations of relevant amplitude.

If electrostatic interactions due to charges are ruled out too, then the activation of dynamics due to iron binding prior to domain closing seems to be possible. The CO_3_^2−^ anion binds in front of the N-terminus of helix 5, which belongs to the domain that moves to close the hinge (residue 91–250, see Additional file 1: Figure S6), before the complex with the iron ion is formed [42]. The residues Tyr92 and His253 contribute to the iron binding and are both located close to the hinge (residue 90–91 and 250–251) [13]. Tyr92 is able to bind to the iron prior to cleft closure, as it is located in the closing domain. We can only speculate that binding of Tyr92 to iron or a similar mechanism may destabilize one strand of the hinge (or both due to hydrogen bonds) and enable large cleft closure movements by reducing the energy difference between open and closed conformation and thus softening the hinge region. This would allow His253 binding for stabilization of the closed cleft. For a short time, until the cleft is stabilized again in the closed conformation, thermal domain motions would be possible, which would not show up in our measurements or structural measurements by X-ray or SANS, but would enable a dynamic cleft closure.

## Methods

### Materials

The molecular mass of human lactoferrin is reported in a range of 75–80 kDa [43, 44], in this work an average value of 77 kDa is used (as in [45]). This value is very close to the calculated mass from the crystal structure of 76.1 kDa. Lactoferrin from human milk was purchased from Sigma-Aldrich, Munich, Germany as lyophilized powder. The protein comes in two different variants: iron saturated (product code L3770) and with no specified iron content (product code L0520). In combination with different buffer conditions, this allows to prepare samples with different iron binding states: Iron-saturated lactoferrin was prepared by dissolving the L3770 powder in a D_2_O-buffer (100 mM 2-(N-morpholino)ethanesulfonic acid (MES), 150 mM NaCl) at pD = 5 and pD = 7. Partially saturated lactoferrin was prepared from L3770, iron-free lactoferrin from L0520, both in an iron-depleting D_2_O-buffer (100 mM MES, 180 mM NaCl, 200 mM NaH_2_PO_4_, 40 mM ethylenediaminetetraacetic acid (EDTA) [9]) at pD = 5. The iron saturation of the protein was measured with light absorption spectroscopy at λ = 465 nm with A^1%^ = 0.58 at an optical path length of 1 mm corresponding to 100 % saturation at a concentration of 1 % weight/volume [5], resulting in 87 %, 30 % and 5 %. The samples were ultra-centrifuged at 150000 g for 2 h to remove larger aggregates. With Dynamic Light Scattering (DLS) measurements the removal of aggregates was verified. The samples were then diluted to the desired concentrations of 2.5, 5, 10 and 50 mg/ml for SANS and NSE measurements. The concentrations were measured with light absorption spectroscopy at λ = 280 nm with an extinction coefficient of e_280_ = 8.85 10^4^ M^−1^cm^−1^ [46]. All experiments mentioned in this work were performed at a temperature of 7 °C to slow down formation of stable aggregates. The release of iron from L3770 (in buffer with EDTA) and iron uptake from iron-free L0520 (in 0.1 M sodium citrate containing FeCl_3_ and NaHCO_3_) showing the expected decrease respectively increase of the 465 nm Fe[III] peak was tested by UV–vis spectroscopy.

#### Small angle neutron scattering

Small Angle Neutron Scattering (SANS) is a technique to study the microscopic structural properties of the sample. The SANS measurements were performed at the instruments KWS1 and KWS2 [47] at the MLZ in Garching, Germany. With a neutron wavelength of λ = 4.5 Å and two detector distances of 1.5 m resp. 2 m and 8 m, a Q-range of about 0.01-0.45 resp. 0.33 Å^−1^ was covered. The wavelength distribution width FWHM was 20 % for KWS2, 10 % for KWS1.

The data reduction to one dimension and merging of the detector distances was done with the instrument-software QtiKWS. The background scattering of the buffer solution was measured separately, scaled with the volume fraction of the buffer and subtracted from the sample signal. The Q-independent incoherent scattering of the protein was calculated from the amino acid sequence, scaled with the protein concentration (e.g. I_inc_(c = 10 mg/ml) = 0.00202 cm^−1^) and subtracted as well (taking into account H/D-exchange between protein and buffer solution [48, 49]). Smearing of the data is taken into account according to Pedersen et al. [50].

The scattering intensity I(Q) of N identical particles is proportional to I(Q) ~ NF(Q)S(Q,c), with the orientational averaged, concentration-independent form factor *F(Q) = <Σ*_*i,j*_ *b*_*i*_ *b*_*j*_ *exp(i*Q*(*r_*i*_ *-*r_*j*_ *))>*, containing information about the shape of the protein and the concentration-dependent structure factor S(Q,c), which represents direct interactions between the proteins in solution. Q is the scattering vector, b_i_ is the coherent neutron scattering length of atom i at position r_i_. For proteins in solution the contrast and H/D exchange have to be considered according to Jacrot [48]. A concentration series allows to extrapolate to c = 0 for each Q-value from the concentration-normalized scattering intensity I(Q)/c to obtain the form factor F(Q) (with S(Q,c = 0) = 1) and to extract the structure factor S(Q,c) successively [19].

The form factor for a protein in solution has an additional contribution from the hydration layer around the protein with a larger density than bulk water [49]. The density of the hydration layer depends on the selected protein and the solvent composition. By a combination of SANS and SAXS the density in the hydration layer can be determined. The main influence for SANS in D_2_O due to the hydration layer is a decrease of the radius of gyration R_g_ and a change in slope at larger Q (e.g. for hLf a reduction of 1 Å for 10 % increase in layer density and the slope flattens significantly). The good reproduction of the hLf form factor without hydration layer in the range 0.07 Å^−1^–0.15 Å^−1^ shows that the change in density for the here examined protein is negligible.

A common issue when dealing with proteins in solution is the formation of aggregates. If the aggregates are sufficiently larger than the single protein molecule, they can be easily detected by dynamic light scattering (DLS) and removed with e.g. centrifugation or filtration. If attractive protein-protein interactions are existent, aggregates will emerge again. However, if the aggregates are similar in size to the protein (R_aggregate_/R_protein_ < 5), they may not be distinguishable any more by DLS and cannot be removed easily. Such a mixture can potentially lead to wrong results in the protein size determination (radius of gyration R_g_) or calculated radial distribution functions as P(r) from SANS data and a false conclusion regarding the conformation.

Our approach is to compare the experimental data with the calculated F(Q) from atomistic protein structures. This provides the opportunity to fathom the reason behind an apparent change in the radius of gyration. Differentiation between conformational changes that alters the protein size and a mixture of single protein molecules and aggregates, both leading to increase in measured radius of gyration R_g,_ is possible. A configurational change e.g. by deformation along a dominant normal mode, which leads to an elongated shape with increased R_g_, will presumably alter the detailed structure visible in F(Q) at larger Q, too. The comparison with the experiment is the criterion to rule out specific deformations. To achieve a protein structure model describing the experimental F(Q) in terms of size (low Q regime) and detailed structure (high Q regime) at the same time, we apply a multi-parameter least square fitting routine. On the one hand, we allow deformations of the protein by e.g. normal modes. On the other hand we enclose additional aggregates and include these in our description by assuming an additional contribution from a Guinier form factor F_agg_(Q) ~ exp(−Q^2^R_agg_^2^/3) for the aggregates. In general the Guinier approximation is valid for QR_g_ < 3^1/2^. To justify the Guinier approximation also for larger Q we compared the scattering of 9 and 10mer metastable clusters and found that the Guinier approximation describes the metastable clusters up to Q = 0.05 Å^−1^ (see Additional file 1: Figure S1).

#### Neutron spin echo spectroscopy

Neutron Spin Echo Spectroscopy (NSE) is a high-resolving quasielastic neutron scattering method to measure the spatial and temporal correlations of scatterers. One obtains directly the normalized intermediate scattering function I(Q,t)/I(Q,0) as the spatial Fourier transform of the Van Hove function G(r,t) [51]. NSE measures coherent and incoherent contribution where the coherent contribution dominates in the SANS regime at low Q. All NSE experiments were performed at the instrument IN15 [52] at the ILL, Grenoble, France. Neutrons with wavelengths of λ = 8, 10, 12 and 16 Å were used, giving an accessible Fourier-time range of up to 200 ns. Only the highest concentration of c = 50 mg/ml of each sample condition was measured. The background contribution of the buffer solution was measured independently and the data for the protein sample was corrected accordingly [19]. Full measurements are shown in the Additional file 1: Figure S3a-c.

#### Modeling diffusion and internal dynamics

The observable single protein diffusion at short times is given by [19]:

1 $$ {D}_0(Q)=\frac{1}{Q^2F(Q)}\left\langle {\displaystyle {\sum}_{j,k}{b}_j{e}^{-iQ{r}_j}}\left(\begin{array}{c}\hfill Q\hfill \\ {}\hfill Q\times {r}_j\hfill \end{array}\right)\boldsymbol{D}\left(\begin{array}{c}\hfill Q\hfill \\ {}\hfill Q\times {r}_k\hfill \end{array}\right){b}_k{e}^{iQ{r}_k}\right\rangle $$

with the form factor F(Q) and the positions r_j_ and scattering lengths b_j_ of the atoms of the protein. The 6x6 diffusion tensor D, the translational diffusion D_0T_ and the rotational diffusion D_0R_, the later two as trace of D can be calculated with the HYDROPRO software from the atomic coordinates [38–40]. The translational diffusion component D_0_(Q = 0) can be verified within the experimental accuracy by DLS using a concentration series and extrapolation to c = 0. The collective diffusion at finite concentration c is given by D(Q) = D_0_(Q)H(Q)/S(Q,c) [53–56]. The effect of the direct interactions is measured in SANS as the structure factor S(Q,c). The solvent-mediated interactions are included in the hydrodynamic function H(Q), which can be assumed to be Q-independent for flexible globular proteins [19] in contrast to rigid globular proteins [57]. From known D_0_(Q) and S(Q,c), the value of H can be determined by DLS and NSE at low Q, where only translational diffusion is observed.

To determine the contribution of internal dynamics quantitatively in the time evolution, we use the equation of Lindsay et al. [58]:

$$ {\left(\frac{I\left(Q,t\right)}{I\left(Q,0\right)}\right)}_{T,R}={e}^{-{Q}^2{D}_T\frac{H_T}{S(Q)}t}{\displaystyle \sum_{l=0}^{\infty }}{S}_l(Q){e}^{-l\left(l+1\right){D}_R{H}_Rt}/{\displaystyle \sum_{l=0}^{\infty }}{S}_l(Q) $$

2 $$ with\ {S}_l(Q)={\displaystyle \sum_m}{\left|{\displaystyle \sum_i}{b}_i{j}_l\left(Q{r}_i\right){Y}_{l,m}\left({\Omega}_i\right)\right|}^2 $$

supplemented for internal dynamics A(Q)e^-Γt^ by Biehl et al. [19]:

3 $$ \frac{I\left(Q,t\right)}{I\left(Q,0\right)}=\left[A(Q){e}^{-\Gamma t}+\left(1-A(Q)\right)\right]\cdot {\left(\frac{I\left(Q,t\right)}{I\left(Q,0\right)}\right)}_{T,R} $$

Thereby, scalar values D_T_ and D_R_ for translational and rotational diffusion are used, as 1/3 of the traces of the respective 3x3 sub-tensors of D from HYDROPRO. The inaccuracy introduced by using two scalars instead of the full tensor can be minimized (<2 %) by rescaling of D_R_ in a way that the initial D_eff_ of [I(Q,t)/I(Q,0)] _T,R_ (for c = 0) matches the calculated D_0_(Q) with the full tensor. The hydrodynamic factor for translation H_T_ is fitted in the low Q regime as described before. The rotational hydrodynamic factor H_R_ is linked to H_T_ and is assumed to have a weaker effect by a factor of 3, as derived by Degiorgio et al. as 1-H_R_ ≈ (1-H_T_)/3 for hard sphere colloids [59]. With the terms S_l_(Q) of a multipole expansion, including the spherical Bessel functions j_l_ and the spherical harmonics Y_l,m_, the protein structure is implicitly included in this calculation. Internal dynamics is described by a Q dependent amplitude A(Q) and an exponential relaxation with relaxation time 1/Γ. Resulting fits to experimental data are shown in Additional file 1: Figure S3a-c.

The contribution A(Q) to the NSE signal of due to thermal fluctuations of the protein structure can be calculated from the displacement vectors for each atom. As a template for the atomic displacements we use elastic normal modes. A(Q) can be calculated from normal mode eigenvectors e^α^ as given by Biehl et al. [17] in approximation for small displacements:

4 $$ A(Q)=\frac{{\displaystyle {\sum}_{\alpha }}{a}_{\alpha }{F}_{\alpha }(Q)}{F(Q)+{\displaystyle {\sum}_{\alpha }}{a}_{\alpha }{F}_{\alpha }(Q)} $$

5 $$ {F}_{\alpha }(Q)=\left\langle {\displaystyle {\sum}_{k,l}^N{b}_k{b}_l{e}^{iQ\left({r}_k-{r}_l\right)}}\left(Q\cdot {e}_k^{\alpha}\right)\left(Q\cdot {e}_l^{\alpha}\right)\right\rangle $$

The eigen frequencies ω_α_ have no real physical meaning, since the normal modes are calculated in vacuum. However, they serve as a measure of stiffness of the respective mode and are used to distribute the thermal energy over all modes equally according to the equipartition theorem. To each normal mode α is assigned a displacement amplitude relative to the first non-trivial mode 6: a_α_ = aω_6_^2^/ω_α_^2^ (modes 0–2 describe translation and modes 3–5 relate to rotation along the three space-axes). Higher frequency modes are suppressed stronger and thus the summation can be limited to the low frequency modes. This leaves the common prefactor *a* as single free parameter.

#### Calculations

Calculations are done under usage of protein structures from the Protein Data Bank (PDB) and the molecular modeling toolkit MMTK [60–62]. SANS form factor calculations and structure refinement by normal modes is done in self-made routines taking into account H/D-exchange as described elsewhere [49] and the contrast of protein scattering with respect to the solvent. Normal modes as motifs for deformation of the unperturbed protein structure were calculated with MMTK using the built-in Amber99 force-field without solvent.

PDB ID: 1lfh, 1b0l, 1dtz

## Additional file

Additional file 1: Metastable aggregates, SANS scattering, form factors, Intermediate scattering function for pD5 and pD7, Effective diffusion, Amplitudes of internal dynamics, Relative displacement amplitudes, relative displacements, N-terminal domain. (PDF 2296 kb)
