# Supplementary material for: Structure and domain dynamics of human lactoferrin in solution and the influence of Fe(III)-ion ligand binding
Source: BMC Biophys. 2016 Nov 4;9:7. doi: 10.1186/s13628-016-0032-3 (PMC5095980; doi:10.1186/s13628-016-0032-3)
Supplement: Additional file 1: — Metastable aggregates, SANS scattering, form factors, Intermediate scattering function for pD5 and pD7, Effective diffusion, Amplitudes of internal dynamics, Relative displacement amplitudes, relative displacements, N-terminal domain. (PDF 2296 kb) [file 13628_2016_32_MOESM1_ESM.pdf]

Structure and domain dynamics of human Lactoferrin in solution and the influence of Fe(III)-ion ligand binding

C. Sill[a,], R. Biehl[a*], B. Hoffmann[b], A. Radulescu[c], M.-S. Appavou[c], B. Farago[d], R. Merkel[b] D. Richter[a]

a JCNS-1 & ICS-1, Forschungszentrum Jülich GmbH, Leo-Brandt Strasse, 52425 Jülich, Germany
b ICS-7, Forschungszentrum Jülich GmbH, Leo-Brandt Strasse, 52425 Jülich, Germany
c JCNS-MLZ, Forschungszentrum Jülich GmbH Outstation at MLZ, Lichtenbergstraße 1 85747 Garching, Germany
d Institut Laue-Langevin, CS 20156, 38042 Grenoble, France

Supplementary Material

Metastable aggregates

The scattering of metastable aggregates cannot be avoided for some protein solutions of hLf. Figure S1a shows the contribution of aggregates to the total scattering including a fit by the Guinier function. Aggregates can be build in silico by placing monomers with a small distance to avoid overlap and to allow a water layer in between the monomers. The scattering of aggregates of 9 and 10 monomers are shown in figure S1b compared to the monomer scattering. The radius of Gyration is 64 Å for the 9 monomer aggregate (see figure S1c) and 67 Å for the 10 monomer aggregate, well within the observed range. Even if the Guinier approximation is not valid for $QR_G>3^{1/2}$ we find a satisfying description up to Q=0.05 $Å^{-1}$. Above Q=0.05 $Å^{-1}$ the contribution from the aggregates is 1-3% of the monomer contribution within this exemplary calculation. To determine the aggregate contribution at Q>0.5 $Å^{-1}$ an ensemble needs to be generated. Within the experimental data the aggregate contribution is below 1% above Q=0.05 $Å^{-1}$.

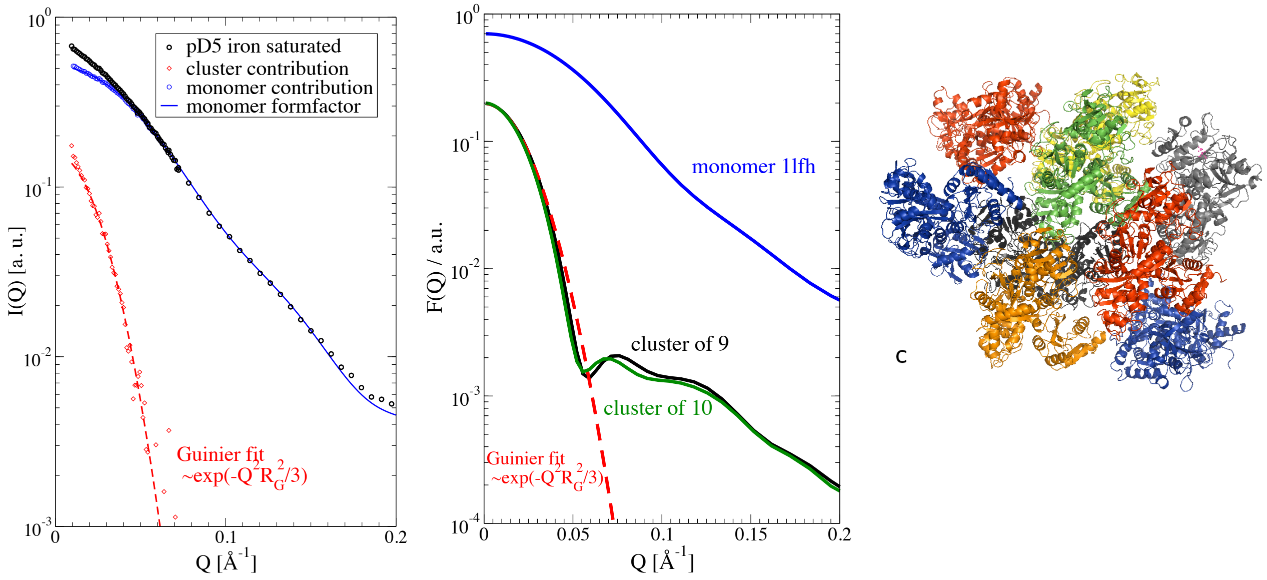

Figure S1 a) Experimental scattering data and decomposition into monomer contribution and aggregate contribution. The monomer formfactor is given as blue line. b) Scattering of exemplary metastable aggregates in comparison to the monomer scattering with respect to the contribution at Q=0 of the experimental data. The dashed line shows the Guinier approximation extrapolated to larger Q. For the 10 monomer aggregate one monomer is added. c) Picture of the 9 monomer aggregate with monomers in different colors.

SANS scattering

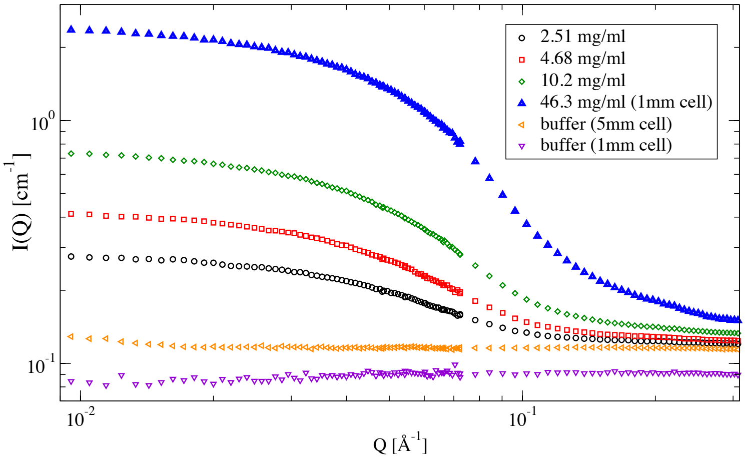

Figure S2a: SANS scattering from a concentration series of iron free hLf. Error bars are smaller than size of the symbols.

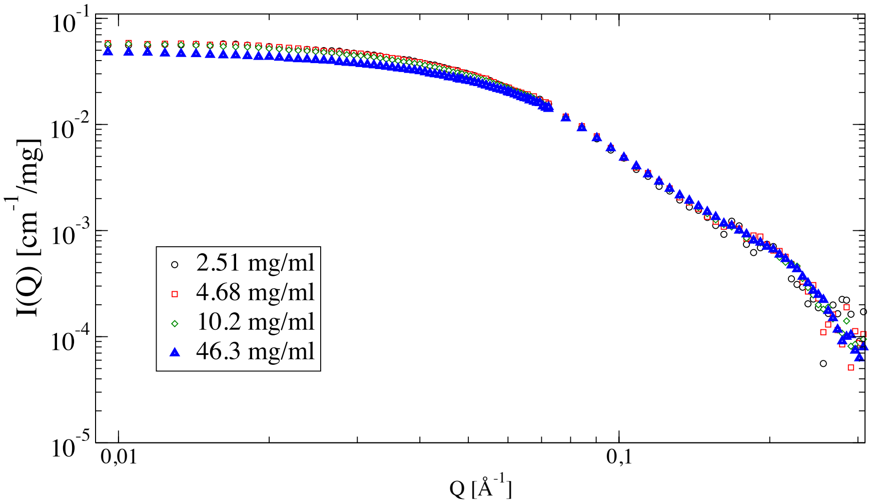

Figure S2b: SANS scattering from a concentration series of iron free hLf after background correction and scaling by concentration. The structure factor is visible as deviation of the highest concentration (blue) with lower values at low Q indicating pure repulsion. Errors for the highest concentration are smaller 0.5% below Q=0.1 Å^{-1} and 2% around 0.2 Å^{-1} (≈symbol size).

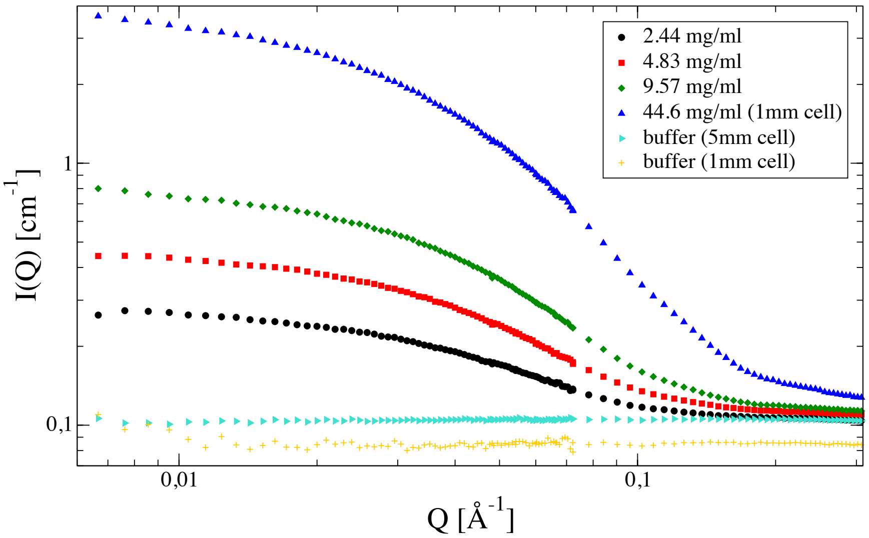

Figure S2c: SANS scattering from concentration series of iron free hLf at pD 5. Errors as above in S2a.

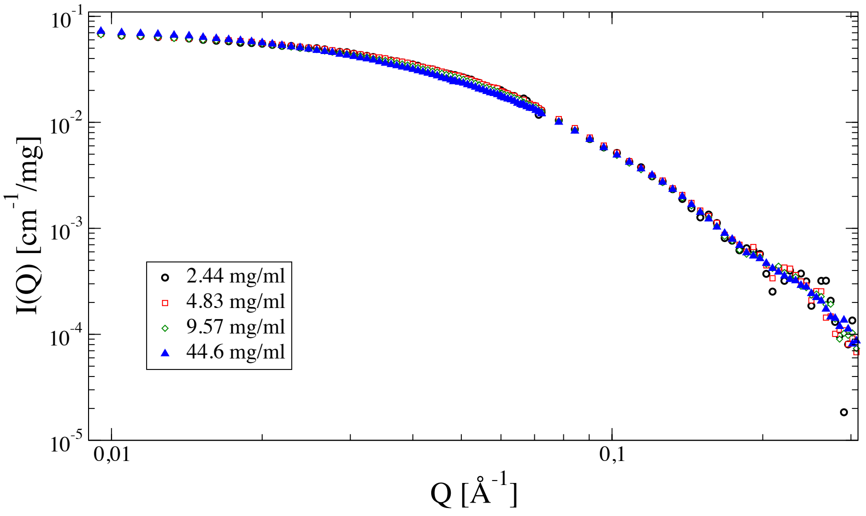

Figure S2d: SANS scattering from concentration series of iron saturated hLf at pD 5 after background correction and scaled by concentration. The structure factor is visible as deviation of the highest concentration (blue). The crossing around 0.02 Å^{-1} indicates that we have an attractive structure factor. Errors for the highest concentration are smaller 0.5% below Q=0.1 Å^{-1} and 2% around 0.2 Å^{-1} (≈symbol size).

Effect of cleft closing on SANS form factor

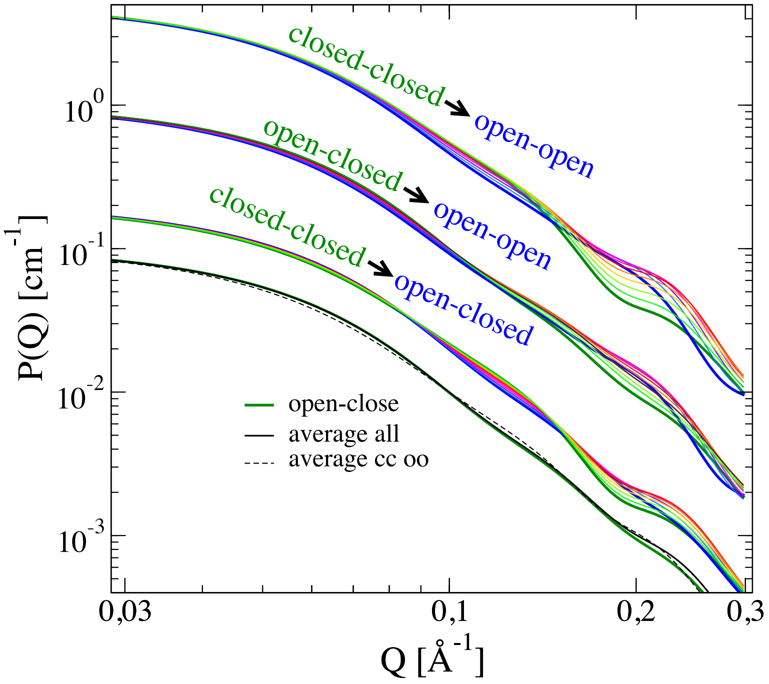

Figure S2e Form factor changes according to the transition from closed to open configurations: closed-closed to open-closed, open-closed to open-open and simultaneous opening of both sides as closed-closed to open-open configuration. Configurations are calculated as linear transition of atom positions from open to closed configurations in 10 steps. Data are shifted by factor 5 up or down for better visibility. The lowest group of lines shows a direct comparison of the open-closed configuration (green) with the average over all configurations

above (dashed black) and the average of open-open and closed-closed configuration (full black, shifted by factor 10).

Intermediate scattering function

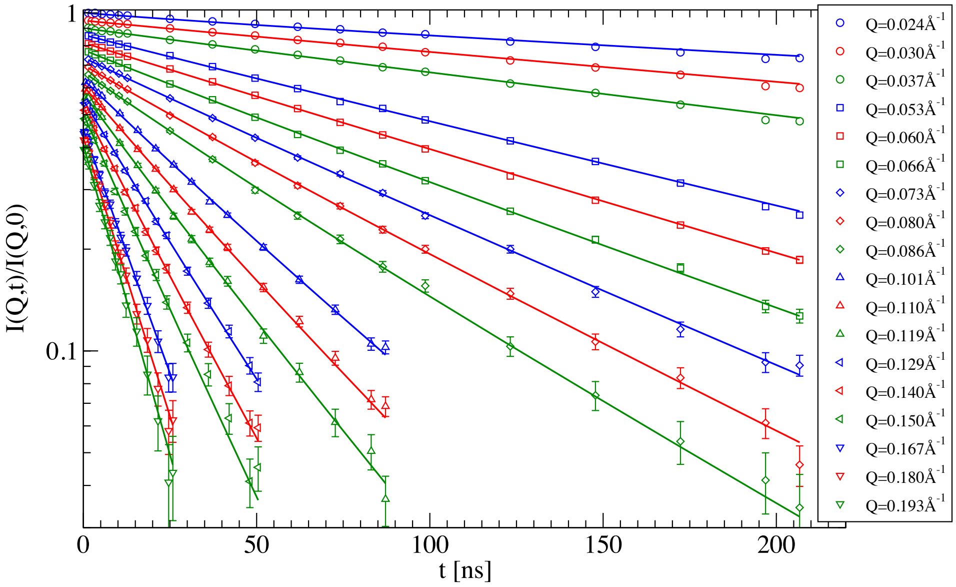

Figure S3a: Intermediate scattering function of iron free hLf at pD 5. 18 Q values were measured with 4 different wavelength and respective time ranges λ = 16 Å : 200ns, λ = 12 Å : 80ns, λ = 10 Å : 50ns. The symbols represent the experimental data, the lines represent the fit according to equ. 3. All data are shifted for better visibility consecutively by a factor 0.95.

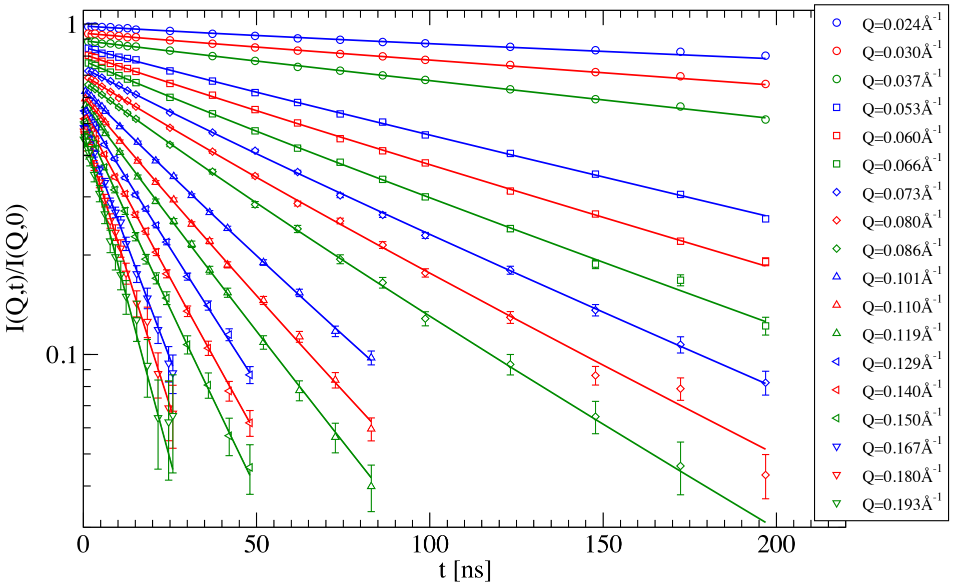

Figure S3b: Intermediate scattering function of iron saturated hLf at pD 5. 18 Q values were measured with 4 different wavelength and respective time ranges λ = 16 Å : 200ns, λ = 12 Å : 80ns, λ = 10 Å : 50ns. The symbols represent the experimental data, the lines represent the fit according to equ. 3. All data are shifted for better visibility consecutively by a factor 0.95.

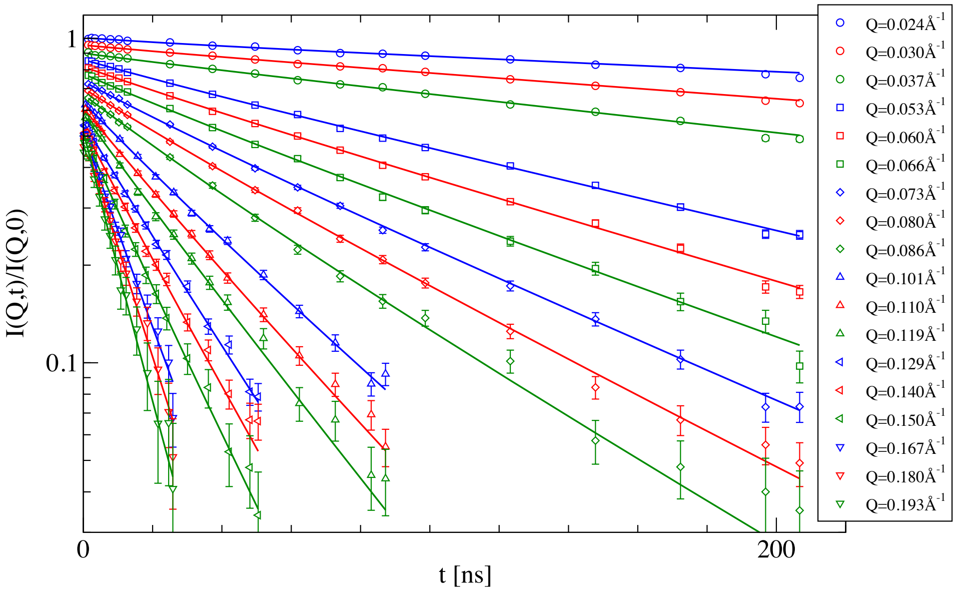

Figure S3c: Intermediate scattering function of iron saturated hLf at pD 7. 18 Q values were measured with 4 different wavelength and respective time ranges λ = 16 Å : 200ns, λ = 12 Å : 80ns, λ = 10 Å : 50ns. The symbols represent the experimental data, the lines represent the fit according to equ. 3. All data are shifted for better visibility consecutively by a factor 0.95.

Effective diffusion

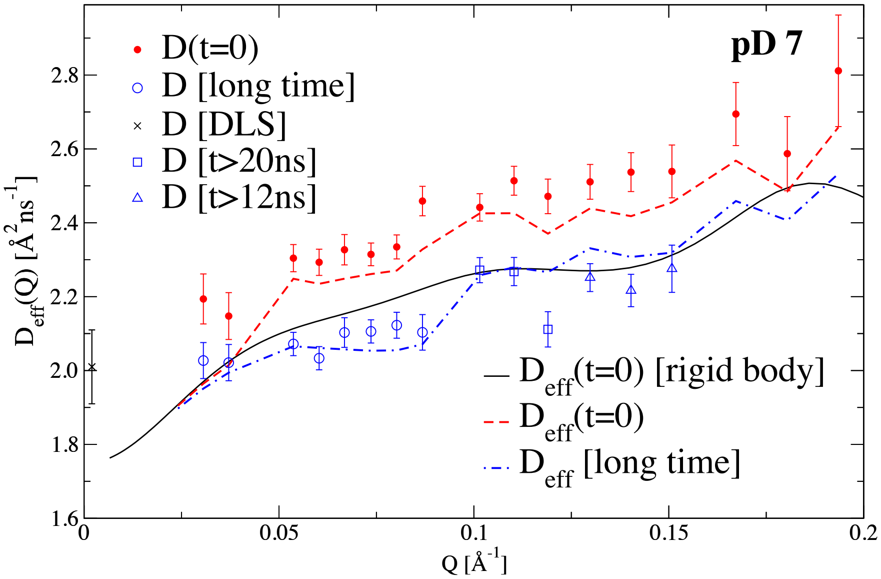

Figure S4 Effective diffusion coefficient Deff of iron saturated sample at pD7: in the initial slope (red dots) and the long time behavior (blue: circles 50ns<t<200ns, squares: 20ns<t<80ns, cross: 12ns<t<50ns). The diffusion constant obtained from DLS (black cross) is shown as well as the expected rigid body diffusion $D_{eff}(Q)=D_0(Q)H/S(Q,c)$ for the open-open model in the initial slope (black line). Broken lines show the slope analysis (initial and long time) of the fitted model, which agrees well with the experimental finding.

Relative displacement amplitudes

	open-open		closed-closed	
Mode	$\omega_6^2 / \omega_\alpha^2$	RMSD [Å]	$\omega_6^2 / \omega_\alpha^2$	RMSD [Å]
7	0.795	5.26	0.817	7.21
8	0.712	4.72	0.643	5.70
9	0.293	1.94	0.369	3.26
10	0.286	1.89	0.263	2.33
11	0.248	1.65	0.243	2.15
12	0.228	1.51	0.164	1.45
13	0.190	1.25	0.154	1.36
14	0.185	1.22	0.140	1.25
15	0.173	1.14	0.130	1.15

Table S5: Relative displacement amplitudes $\omega_6^2 / \omega_\alpha^2$ and root mean square displacements for the normal modes of the open-open and closed-closed structures. RMSD's are calculated as $\left\langle \sqrt{a_\alpha} \mathbf{e}^\alpha \right\rangle$ with the mode amplitudes a_α corresponding to the fitted A(Q). The RMSD of mode 6 corresponding to the higher modes in the later table is 6.6 Å (open-open) and 8.8 Å (closed-closed) configuration.

N-terminal domain

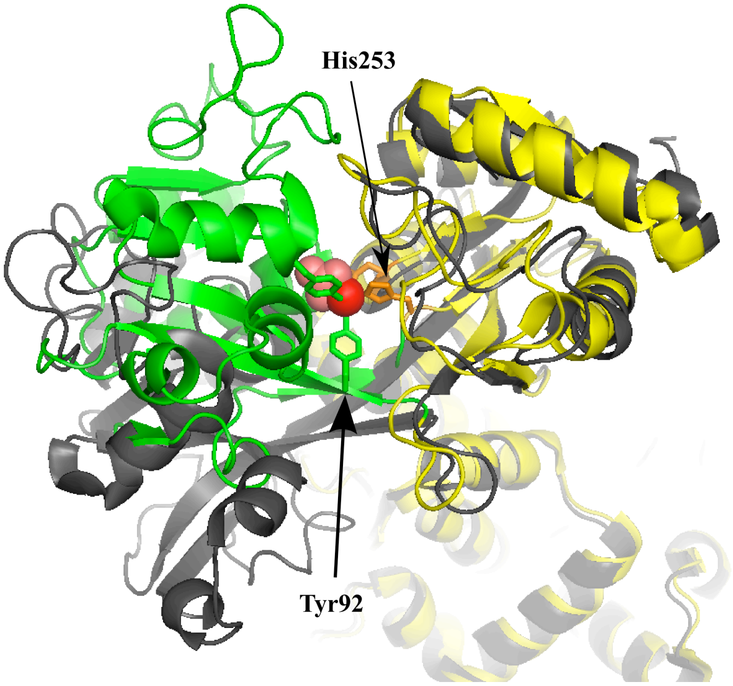

Figure S6: N-terminal lobe binding site. Iron free Lf (PDB: 1lfh in dark grey) aligned to iron bound Lf (PDB: 1b0l in yellow and green). The cleft closing domain is marked in green reaching from residue 91 to residue 250. The iron and the anion are shown as red, respectively light red spheres. The binding amino acids are shown as sticks in green and orange. Tyr192 and His 253 are close to the hinge between yellow and green colored 1b0l domains.
